# Cannabis use in youth is associated with chronic inflammation

**DOI:** 10.1017/S0033291724002848

**Published:** 2024-12

**Authors:** Emmet Power, David Mongan, Colm Healy, Subash Raj Susai, Melanie Föcking, Stanley Zammit, Mary Cannon, David Cotter

**Affiliations:** 1 Department of Psychiatry, Royal College of Surgeons in Ireland, Smurfit Building, Beaumont Hospital, Dublin 9, Ireland; 2 Department of Liaison Psychiatry, Children's Health Ireland, Dublin 1, Ireland; 3 Centre for Public Health, Queens University Belfast, Belfast, UK; 4 Centre for Clinical Brain Sciences, Division of Psychiatry, University of Edinburgh, Edinburgh, UK; 5 Department of Child and Adolescent Psychiatry, School of Medicine, University College Dublin, Dublin, Ireland; 6 Centre for Academic Mental Health, Population Health Sciences, Bristol Medical School, University of Bristol, Bristol, UK; 7 Division of Psychological Medicine and Clinical Neurosciences, MRC Centre for Neuropsychiatric Genetics and Genomics, Cardiff University School of Medicine, Cardiff, UK; 8 Department of Liaison Psychiatry, Beaumont Hospital, Dublin 9, Ireland; 9 FutureNeuro Research Ireland Centre, Royal College of Surgeons in Ireland, Smurfit Building, Beaumont Hospital, Dublin 9, Ireland

**Keywords:** ALSPAC, cannabis, endocannabinoid, inflammation, suPAR, youth

## Abstract

**Background:**

Markers of inflammation and cannabis exposure are associated with an increased risk of mental disorders. In the current study, we investigated associations between cannabis use and biomarkers of inflammation.

**Methods:**

Utilizing a sample of 914 participants from the Avon Longitudinal Study of Parents and Children, we investigated whether interleukin-6 (IL-6), tumor necrosis factor *α* (TNF*α*), C-reactive protein (CRP), and soluble urokinase plasminogen activator receptor (suPAR) measured at age 24 were associated with past year daily cannabis use, less frequent cannabis use, and no past year cannabis use. We adjusted for a number of covariates including sociodemographic measures, body mass index, childhood trauma, and tobacco smoking. We found evidence of a strong association between daily or near daily cannabis use and suPAR.

**Results:**

We did not find any associations between less frequent cannabis use and suPAR. We did not find evidence of an association between IL-6, TNF*α* or CRP, and cannabis use.

**Conclusions:**

Our finding that frequent cannabis use is strongly associated with suPAR, a biomarker of systemic chronic inflammation implicated in neurodevelopmental and neurodegenerative processes is novel. These findings may provide valuable insights into biological mechanisms by which cannabis affects the brain and impacts the risk of serious mental disorders.

## Introduction

The impact of cannabis use on the development of serious and enduring mental illness has been a focus of research for over 120 years (Power, Healy, Murray, & Cannon, 2023). Cannabis use in youth has been linked to a range of deleterious neurodevelopmental outcomes including accelerated cortical thinning, declines in verbal intelligence and the development of psychotic disorders (Albaugh et al., 2023; Power et al., 2021, 2023). A recent umbrella review concluded that childhood trauma and cannabis use are the only two potentially modifiable risk factors associated with later psychosis that have strong prospective evidence (Arango et al., 2021). The potential mechanisms linking early cannabis use to later psychosis remain poorly understood although epidemiological evidence increasingly points toward the association being causal (Gage et al., 2017; Power et al., 2023). There are some conflicting lines of evidence within the available literature concerning long-term cannabis use and psychosis. Chronic cannabis use has been associated with decreased synthesis of dopamine in the striatum while increased dopamine synthesis in the striatum is generally accepted to be a neurobiological substrate of acute psychotic episodes (Bloomfield, Ashok, Volkow, & Howes, 2016; Murray et al., 2017).

More recently, hypotheses have emerged implicating inflammation as a mechanism in the development of serious mental illness such as psychosis (Mongan, Ramesar, Föcking, Cannon, & Cotter, 2020). Mongan et al. (2020) in their review highlight that disruptions to the blood–brain barrier, activation of microglia, and abnormalities in the complement system may be implicated in excessive dendritic pruning characterized in psychotic disorders. In the case of cannabis use, the implication of neuroimmune function is of specific interest. Several lines of preclinical evidence from animal models show a persistent neuroinflammatory state resulting from adolescent Δ9-tetrahydrocannabinol (THC) exposure (De Meij et al., 2021; Moretti et al., 2014, 2015; Zamberletti, Gabaglio, Prini, Rubino, & Parolaro, 2015). These studies have suggested an effect from THC exposure in adolescence to later persistent neuroinflammation in adulthood and that THC may cause aberrations in neuroimmune development which later impact neurological integrity. One further recent study found that even low dose chronic THC treatment in adolescence resulted in increases in microglia expressing activated states associated with inflammatory processes in the brain and that these microglial changes persisted to adulthood (Lee et al., 2022). In this study, Lee and colleagues also found an enhanced neuroimmune response to subsequent social defeat stress including enhanced recruitment of peripheral monocytes to the brain leading to further microglia activation (Lee et al., 2022). The model described by Lee et al. (2022) is of specific interest as previous epidemiological evidence has suggested that severe stressors may moderate the risk of psychosis substantially in early onset cannabis users (Harley et al., 2010; Houston, Murphy, Adamson, Stringer, & Shevlin, 2008). A summary of both these studies showed that early onset cannabis use and severe stressors interact to increase the relative risk of subsequent psychosis more than 14-fold (Kiburi, Molebatsi, Ntlantsana, & Lynskey, 2021). These findings converge with evidence that cumulative stressful life events are related to peripheral soluble urokinase plasminogen activator receptor (suPAR), a marker of systemic chronic inflammation, in a dose–response manner (Bourassa et al., 2021).

Daily cannabis use is known to be associated with downregulation of cannabinoid receptor 1 (CB1R) in a regionally specific manner, affecting the neocortex and limbic cortex but not subcortical structures or the cerebellum (Hirvonen et al., 2012). The relationship between CB1R and inflammation in the periphery has been described in animal models (Han & Kim, 2021).

Previous studies have showed mixed results, however, with some suggesting that the endocannabinoid system exerts anti-inflammatory effects (Giacobbe, Marrocu, Di Benedetto, Pariante, & Borsini, 2021; Pacher, Steffens, Haskó, Schindler, & Kunos, 2018). A recent transcriptomic study has highlighted that the effect of THC on inflammation-related gene expression is nuanced and is not solely immunosuppressive (Hu et al., 2020). Pro-inflammatory effects may be related to dose, developmental sensitivity in timing of exposure, tissue type, and duration of exposure (Li, Watkins, & Wang, 2024; Moretti et al., 2014, 2015; Pacher et al., 2018; Zamberletti et al., 2015).

A number of studies in humans investigating cannabis and biomarkers of inflammation have been carried out. Da Silva et al. (2019) used positron emission tomography (PET) imaging to measure *in vivo* brain levels of translocator protein (TSPO), an indicator of neuroinflammation, hypothesizing to find reduced TSPO in cannabis users. They found long-term cannabis users exhibited significantly higher TSPO levels in several brain regions compared to non-users. In contrast to their initial hypothesis they found increased brain TSPO with effect sizes of medium to large (Cohen's *d* = 0.6) and large (Cohen's *d* = 0.8) magnitudes in contrast to controls. A further PET study by the same group in a different set of samples replicated these findings showing similar effect-size associations between genetically predicted Complement component 4a protein (C4a) and TSPO, and cannabis use and TSPO (Da Silva et al., 2021). Notably both C4a and cannabis use are suggested to be implicated in the development of psychosis. Fernandez-Egea et al. (2013) found increased peripheral serum levels of C-C motif chemokine 11 (CCL11) in current cannabis users independent of tobacco use. CCL11 has previously been associated with neuroinflammatory processes, aging, and schizophrenia. A limited number of studies have investigated relationships between cannabis use and other peripheral inflammatory markers (interleukin-6 [IL-6], fibrinogen, C-reactive protein [CRP], IL-1*β*, and tumor necrosis factor *α* [TNF*α*]) in population samples (Corsi-Zuelli et al., 2022; Ferguson, Mannes, & Ennis, 2019; Okafor, Li, & Paltzer, 2020). These studies found no evidence of a direct association. Okafor et al. (2020) and Ferguson et al. (2019) measured past 30 day use as an exposure which may include many infrequent users. Corsi-Zuelli et al. (2022) found that inflammation may moderate the association between cannabis use and psychosis in early onset daily users, but found no linear association between cannabis use and inflammatory markers overall.

No studies to date have investigated associations between cannabis use and suPAR. suPAR is known to be extensively implicated in neurodevelopmental and neurodegenerative processes, and innate immune responses in the brain (Cunningham et al., 2009). suPAR in serum is correlated with both cerebrospinal fluid (CSF) and albumin (Garcia-Monco, Coleman, & Benach, 2002). Elevated suPAR is found in an array of neurological and neuropsychiatric diseases (Garcia-Monco et al., 2002; Murphy et al., 2024). suPAR may also represent a more reliable measure of systemic chronic inflammation as it is less influenced by potential confounding factors such as intercurrent infection, medication use, and diurnal variation compared to IL-6 and CRP which are acute phase reactants (Rasmussen, Petersen, & Eugen-Olsen, 2021). suPAR is known to have high temporal stability (Rasmussen et al., 2021). suPAR is also hypothesized to predict subclinical end-organ damage and cellular inflammation whereas CRP is differentially hypothesized to predict metabolic inflammation (Lyngbæk et al., 2013).

In the present study we aimed to investigate in a population sample whether cannabis use, characterized as daily/near daily use or other frequencies of past year use, was associated with measures of IL-6, TNF*α*, CRP, and suPAR. CB1R agonism is not associated with adverse metabolic or anthropometric changes in humans but is associated with altered mitochondrial respiration (Athanasiou et al., 2007; Meier et al., 2016; Meier, Pardini, Beardslee, & Matthews, 2019). In line with Lyngbæk et al. (2013) on the cellular *v.* metabolic inflammation differentiation hypothesis between suPAR and CRP: we hypothesized that elevated suPAR would be associated with cannabis use, whereas elevated CRP and related acute phase reactants, IL-6 and TNF*α* would not be associated with cannabis use.

## Methods

### Cohort profile

The Avon Longitudinal Study of Parents and Children (ALSPAC) birth cohort is a longitudinal cohort study that enrolled 14 541 pregnant women resident in a defined geographical region in southwest England with expected dates of delivery between April 1st, 1991 and December 31st, 1992. From birth of the study subject, parents participated in regular surveys of the study subject's health and environment. From age 7 the children attended periodic assessment clinics in which they participated in clinical interviews and underwent physical tests. In total, 9958 participants were invited to attend clinic at the age 24, of whom 4019 attended the clinic and 3257 of whom provided a blood sample (Boyd et al., 2013; Fraser et al., 2013; Northstone et al., 2019). Ethical approval was received from the ALSPAC Law and Ethics Committee. Participants provided written informed consent and there was no financial compensation. For this analysis, we obtained local ethical approval from the Royal College of Surgeons in Ireland (REC1240cc). Consent for biological samples was collected in accordance with the Human Tissue Act 2004. Data were collected and managed using REDCap data capture tools (Harris et al., 2009, 2019). Note that the study website contains details of all the data that are available through a fully searchable data dictionary and variable search tool (http://www.bristol.ac.uk/alspac/researchers/our-data/).

We report on a subgroup of 914 from the 3257 individuals who attended the age 24 clinic and provided a blood sample. Of the 914 individuals selected for analysis, 22.1% reported major depressive disorder, 29.32% reported generalized anxiety disorder, and 9.74% reported any past 6 month history of any psychotic symptoms.

### Inflammatory markers

ALSPAC collected venous blood samples using a standardized protocol during a clinic visit when participants were age 24. Prior to phlebotomy, participants were requested to fast for 6 h. Samples were immediately spun and the plasma was separated and frozen at −80°C. Samples were subjected to one freeze–thaw cycle for aliquoting immediately prior to performing TNF*α*, CRP, and IL-6 assays.

We analyzed the age 24 subsample with multiplex immunoassay kits. Plasma concentrations of TNF*α* and IL-6 were measured using the Proinflammatory Panel 1 (human) and CRP using Vascular Injury Panel 2 (human) kits (Meso Scale Discovery). For these markers, samples were measured in duplicate and we excluded cases from analysis where the coefficient of variation was >20% for quality control purposes.

Plasma concentrations of suPAR were measured using a suPARnostic ELISA kit (Virogates) according to the manufacturer's instructions. A SpectraMax M3 microplate reader was used to measure optical densities. A standard curve was generated for each plate and plasma concentrations were interpolated using Virogates' custom results calculation tool (https://www.virogates.com/support). Standards were measured in duplicate and participant samples in singlet. All biomarker numerical outcome data are standardized to a mean of 0 and where 1 unit is equal to a 1 standard deviation (s.d.).

### Cannabis use

Cannabis use frequency was measured by a single question with a number of response options for past year use. We grouped these responses into three coded options: (1) daily/almost daily use, (2) weekly or monthly use, (3) less frequent past year use, or (4) no use (representing the dummy variable). We chose to categorize cannabis as such to reflect likely types of use (non-use, experimental, recreational, and dependent).

### Covariates

We adjusted for a range of covariates in a sequential manner aligned with previous described methods (O'Connor et al., 2009). We present unadjusted results, followed by a model adjusted for smoking status, body mass index (BMI), sex, and age in months. The third model is adjusted for early life sociodemographic measures (maternal education and family income), current alcohol dependency symptoms, and childhood trauma. The final model is adjusted for concurrent psychiatric comorbidity (generalized anxiety, moderate-to-severe depression, and experiencing psychotic symptoms in the past 6 months).

BMI was measured at clinic visits using a wall-mounted stadiometer and electronic scales. Smoking status was coded as no past 30 day smoking, non-daily smoking in the past 30 days, and daily smoking. Depressive and anxiety disorders were measured by the Clinical Interview Schedule-Revised (CIS-R) at the age 24 ALSPAC clinic visit. The CIS-R classifies generalized anxiety disorder diagnosis as well as depression as mild, moderate, or severe based on symptom counts. The CIS-R instrument is a high specificity screening tool administered by trained lay interviewers in a computerized format (Brugha et al., 1999). We coded depression as a binomial variable with moderate-to-severe depression coded as 1 and mild and no depression coded as zero. Psychotic symptoms were measured using the PLIKSi which is a 12-item scale of measure of positive and thought interference symptoms of psychosis. This measure was rated by trained psychologists, where symptoms are rated as suspect, definite, or none. We coded symptoms as positive if their occurrence was definite, in the past 6 months and not attributable to sleep or fever (Horwood et al., 2008). Alcohol use was measured with the Alcohol Use Disorders Identification Test-Consumption (AUDIT-C) score. The AUDIT-C score performs similarly to the full AUDIT screening tool in detecting hazardous drinking and/or alcohol abuse or dependence (Bush, Kivlahan, McDonell, Fihn, & Bradley, 1998). We utilized a previously described trauma classification within ALSPAC compiled from a mixture of contemporaneous subject and parent reports and age 22 subject retrospective reports (Croft et al., 2019). We selected five trauma variables including emotional neglect, emotional abuse, physical abuse, sexual abuse, and exposure to domestic violence. These variables were measured by time of exposure, from 0 to 4.9 years, 5 to 11.9 years, and 12 to 17.9 years. We coded each trauma exposure by summing the number of time point exposures. The range was 0–3 except for emotional neglect coded as 0–2 as it was not measured between 0 and 4.9 years.

### Statistical analysis

We used Stata version 18 for statistical analysis. We log_e_-transformed values for suPAR, TNF*α*, IL-6, and CRP prior to analysis to account for skewness. We winsorized any outlying values to +/- 4 standard deviations. We used multivariate linear regression analyses to examine associations between endorsing cannabis use frequency and CRP, IL-6, and TNF*α*. We used multiple linear regression to investigate associations between past year cannabis use frequency and suPAR. We analyzed suPAR separately to other biomarkers as it is reportedly not an acute phase reactant and is proposed to reflect different aspects of inflammation (Lyngbæk et al., 2013).

We used multiple imputation with chained equations to impute missing independent variable and covariate data with the *ice* package in Stata. We imputed 100 datasets for analyses for markers of missing exposure and covariate data separately (Graham, Olchowski, & Gilreath, 2007; Royston, 2014). We imputed for missing data using 35 variables to make the missing at random assumption more plausible. These included data points of parental income during the subject's gestation and childhood, the subject's substance and alcohol use history, mental health outcome data, parental home ownership, maternal post-natal depression, residency status (i.e. living with parents, in rented or owned accommodation), and current participation in employment and/or training. We used the *mim* package to apply Rubin's rules (Rubin, 1976, 2004). We report complete case analysis within our online Supplementary materials.

## Results

Of the 914 subjects whose samples underwent suPAR measurements, 913 samples passed quality control. For the multiplex assay results, a lower number of samples passed quality control for all three biomarkers, totaling 767 samples.

In Table 1 we provide the sample characteristics of the complete sample.

**Table 1.** Sample characteristics

	%	% missing
*Exposures*		
% Less than monthly use	20.97	0.33
% Weekly/monthly cannabis use	6.89	0.33
% Daily cannabis use	4.7	0.33
*Covariates*		
Emotional abuse (%)	20.89	12.04
Emotional neglect (%)	7.33	15.54
Physical abuse (%)	22.77	8.86
Childhood sexual abuse (%)	12.58	27.38
Domestic violence (%)	13.89	17.2
Mean BMI (s.d.)	24.83 (5.3)	0
Past-month non-daily smoking	17.51	0.77
Daily smoking	14.22	0.77
Median AUDIT-C score (IQR)	5 (4–7)	1.64
Female	62.25	0
Mean age in months (s.d.)	294.1 (9.4)	0
% Low-income household in pregnancy	1.2	24.64
Maternal education – degree level	22.56	8.65
Moderate/severe depression	22.1	0.66
Generalized anxiety	29.32	0.44
Psychotic symptoms in last 6 months	9.74	0.88

The prevalence of tobacco smoking was relatively high; 17.51% were non-daily past month smokers, and 14.22% were current daily smokers. Mean BMI was 24.83. Median and interquartile values of AUDIT-C scores were 5 and 4–7. There was little evidence of severe early life financial disadvantage. In total, 1.2% had a weekly household income of <100 pounds sterling during their gestation. Approximately 22.56% of subjects in both subgroups had mothers who were holders of university degrees during their gestation. Physical abuse (22.77%) and emotional abuse (20.89%) were the most common forms of childhood trauma reported. Other abuse types were less prevalent; 13.89% reported exposure to domestic violence, 7.33% reported emotional neglect, and 12.58% reported childhood sexual abuse. Daily use of cannabis was reported in 4.7%, weekly to monthly cannabis use was reported by a further 6.89%, and past year use which was less than monthly was reported by 20.97%.

Sample characteristics did not substantially within complete case analysis (see online Supplementary eTable 1).

### IL-6, CRP, and TNF*α* findings

In Tables 2–4,, we show the results of our findings investigating cannabis use and IL-6, CRP, and TNF*α*.

**Table 2.** Multivariate regression analyses of associations between log CRP, log IL-6, log TNF*α*, and cannabis use frequencies in 100 imputed samples (results for log CRP)^a^

Frequency of cannabis use	Inflammatory marker			
	Log CRP			
Model 1	*β*	CI	*p* value	FMI
<Monthly use	−0.073	−0.247 to 0.102	0.415	0.001
Weekly/monthly use	0.002	−0.275 to 0.28	0.987	0.001
Daily use	−0.179	−0.528 to 0.17	0.315	0.001
Model 2	*β*	CI	*p* value	FMI
<Monthly use	−0.002	−0.176 to 0.171	0.981	0.007
Weekly/monthly use	0.135	−0.134 to 0.405	0.325	0.004
Daily use	−0.099	−0.447 to 0.248	0.575	0.012
Model 3	*β*	CI	*p* value	FMI
<Monthly use	0.021	−0.156 to 0.199	0.812	0.01
Weekly/monthly use	0.149	−0.125 to 0.423	0.287	0.011
Daily use	0.116	−0.47 to 0.239	0.522	0.021
Model 4	*β*	CI	*p* value	FMI
<Monthly use	0.038	−0.141 to 0.214	0.684	0.01
Weekly/monthly use	0.175	−0.101 to 0.451	0.213	0.011
Daily use	−0.078	−0.436 to 0.28	0.668	0.021

CRP, C-reactive protein; FMI, fraction of missing information; CI, confidence interval.

a Each 1-unit increase in the *β* coefficient is equivalent to a 1 s.d. increase. Model 1 is unadjusted. Model 2 is adjusted for age in month, sex, tobacco use, and BMI. Model 3 is additionally adjusted for maternal education in pregnancy, weekly family income in pregnancy, and AUDIT-C score. Model 4 is additionally adjusted for generalized anxiety disorder, moderate-to-severe depressive disorder, and definite psychotic experiences in the last 6 months.

**Table 3.** Multivariate regression analyses of associations between CRP, IL-6, TNF*α*, and cannabis use frequencies in 100 imputed samples (results for IL-6)^a^

Frequency of cannabis use	Inflammatory marker			
	Log IL-6			
Model 1	*β*	CI	*p* value	FMI
<Monthly use	−0.073	−0.247 to 0.102	0.415	0.001
Weekly/monthly use	0.002	−0.275 to 0.28	0.987	0.001
Daily use	−0.179	−0.528 to 0.17	0.315	0.001
Model 2	*β*	CI	*p* value	FMI
<Monthly use	0.015	−0.156 to 0.187	0.861	0.008
Weekly/monthly use	0.239	−0.027 to 0.506	0.079	0.010
Daily use	−0.054	−0.399 to 0.289	0.754	0.021
Model 3	*β*	CI	*p* value	FMI
<Monthly use	0.037	−0.137 to 0.212	0.677	0.012
Weekly/monthly use	0.257	−0.014 to 0.527	0.063	0.016
Daily use	−0.089	−0.44 to 0.262	0.619	0.035
Model 4	*β*	CI	*p* value	FMI
<Monthly use	0.033	−0.142 to 0.208	0.710	0.012
Weekly/monthly use	0.255	−0.018 to 0.527	0.067	0.016
Daily use	−0.117	−0.472 to 0.238	0.519	0.031

IL-6, interleukin 6; FMI, fraction of missing information; CI, confidence interval.

a Each 1-unit increase in the *β* coefficient is equivalent to a 1 s.d. increase. Model 1 is unadjusted. Model 2 is adjusted for age in month, sex, tobacco use, and BMI. Model 3 is additionally adjusted for maternal education in pregnancy, weekly family income in pregnancy, and AUDIT-C score. Model 4 is additionally adjusted for generalized anxiety disorder, moderate-to-severe depressive disorder, and definite psychotic experiences in the last 6 months.

**Table 4.** Multivariate regression analyses of associations between CRP, IL-6, TNF*α*, and cannabis use frequencies in 100 imputed samples (results for TNF*α*)^a^

Frequency of cannabis use	Inflammatory marker			
	Log TNF*α*			
Model 1	*β*	CI	*p* value	FMI
<Monthly use	0.02	−0.156 to 0.196	0.822	0.006
Weekly/monthly use	−0.133	−0.412 to 0.147	0.353	0.004
Daily use	−0.18	−0.533 to 0.172	0.316	0.009
Model 2	*β*	CI	*p* value	FMI
<Monthly use	−0.023	−0.213 to 0.167	0.811	0.008
Weekly/monthly use	−0.213	−0.508 to 0.082	0.156	0.004
Daily use	−0.326	−0.706 to 0.054	0.093	0.012
Model 3	*β*	CI	*p* value	FMI
<Monthly use	−0.014	−0.207 to 0.18	0.890	0.012
Weekly/monthly use	−0.202	−0.502 to 0.097	0.186	0.010
Daily use	−0.344	−0.731 to 0.044	0.082	0.024
Model 4	*β*	CI	*p* value	FMI
<Monthly use	−0.024	−0.219 to 0.17	0.807	0.013
Weekly/monthly use	−0.216	−0.518 to 0.085	0.160	0.009
Daily use	−0.389	−0.781 to 0.003	0.052	0.021

TNF*α*, Tumor necrosis factor *α*; FMI, fraction of missing information; CI, confidence interval.

a Each 1-unit increase in the *β* coefficient is equivalent to a 1 s.d. increase. Model 1 is unadjusted. Model 2 is adjusted for age in month, sex, tobacco use, and BMI. Model 3 is additionally adjusted for maternal education in pregnancy, weekly family income in pregnancy, and AUDIT-C score. Model 4 is additionally adjusted for generalized anxiety disorder, moderate-to-severe depressive disorder, and definite psychotic experiences in the last 6 months.

No type of exposure to cannabis use was associated with CRP, IL-6, or TNF*α*.

### suPAR findings

We outline the findings of our analyses investigating associations between cannabis use and suPAR in Table 5.

**Table 5.** Linear regression analyses of associations between suPAR and past year cannabis use frequencies in 100 imputed samples^a^

Frequency of cannabis use	Inflammatory marker			
	Log suPAR			
Model 1	*β*	CI	*p* value	FMI
Less than monthly use	−0.108	−0.255 to 0.039	0.15	0.034
Weekly/monthly use	0.024	−0.209 to 0.257	0.841	0.022
Daily use	0.651	0.372–0.93	<0.001	0.03
Model 2	*β*	CI	*p* value	FMI
Less than monthly use	−0.064	−0.212 to 0.084	0.400	0.037
Weekly/monthly use	0.054	−0.176 to 0.285	0.644	0.025
Daily use	0.575	0.289–0.861	<0.001	0.021
Model 3	*β*	CI	*p* value	FMI
Less than monthly use	−0.018	−0.168 to 0.133	0.819	0.04
Weekly/monthly use	0.069	−0.164 to 0.301	0.562	0.026
Daily use	0.522	0.232–0.811	<0.001	0.03
Model 4	*β*	CI	*p* value	FMI
Less than monthly use	−0.032	−0.183 to 0.118	0.675	0.04
Weekly/monthly use	0.032	−0.201 to 0.265	0.786	0.027
Daily use	0.474	0.183–0.765	0.001	0.03

suPAR, soluble urokinase plasminogen activator receptor; FMI, fraction of missing information; CI, Confidence interval.

a Each 1-unit increase in the *β* coefficient is equivalent to a 1 s.d. increase. Model 1 is unadjusted. Model 2 is adjusted for age in month, sex, tobacco use, and BMI. Model 3 is additionally adjusted for maternal education in pregnancy, weekly family income in pregnancy, and AUDIT-C score. Model 4 is additionally adjusted for generalized anxiety disorder, moderate-to-severe depressive disorder, and definite psychotic experiences in the last 6 months.

We observed a significant association of suPAR with daily or near daily cannabis use (*β* = 0.474, confidence interval [CI] 0.183–0.765; *p* = 0.001). In contrast, suPAR was not associated with weekly/monthly cannabis use (*β* = 0.032, CI −0.201 to 0.265; *p* = 0.786) or less frequent past year cannabis use (*β* = −0.032, CI −0.183 to 0.118; *p* = 0.675).

The results of our complete case analysis did not significantly differ from the findings of our imputed analyses and can be viewed in the online Supplementary materials (see eTables 2 and 3).

## Discussion

We found novel evidence that current daily/near daily cannabis use was associated with elevated suPAR in youth in this sample at mean age 24. There was strong evidence for the association between daily/near daily cannabis use and elevated suPAR (*p* = 0.001). Even when applying a Bonferroni correction given there were two models specified in this study, this result remains highly significant at *p* = 0.002. Compared to non-users, daily cannabis users had elevated levels of serum suPAR compared to controls of approximately 0.5 s.d. when confounding factors are extensively accounted for.

Our findings of a strong association between daily/near daily cannabis use and elevated suPAR are in line with animal experimental studies which investigated the association of Δ9-THC, the psychoactive component of cannabis, with inflammation (Moretti et al., 2014; Zamberletti et al., 2015). To our knowledge this is the first study to examine associations between cannabis use and suPAR.

The impact of cannabis use on health, particularly in terms of risk cardiovascular events and atherosclerosis, has been well-described (Cheung, Coates, Millar, & Burr, 2021; Pacher et al., 2018). Our findings, specifically the association between suPAR and daily cannabis use, are along some conflicting lines of evidence around the association between cannabis use and chronic disease morbidity. To date longitudinal studies have not demonstrated evidence of independent associations between heavy cannabis use and later chronic disease morbidity conclusively as heavy cannabis users tend to engage in other health risk behaviors (Meier et al., 2016, 2019, 2022). These studies however have contained relatively small numbers of long-term cannabis users. Large register-based observational studies have recorded excessive morbidity and mortality associated with cannabis use disorder; however, a causal relationship between cannabis use and excessive mortality is questioned by some authors (Calabria, Degenhardt, Hall, & Lynskey, 2010; Hoch et al., 2024; Weye et al., 2020). Our study provides a plausible mechanism by which cannabis may be related to chronic inflammation, which is implicated in chronic disease. suPAR is known to implicated in a variety of chronic diseases and predicts mortality in the general population, therefore our findings support cannabis use being implicated in excess mortality (Haupt et al., 2019; Petersen, Kallemose, Barton, Caspi, & Rasmussen, 2020). To what extent the association between cannabis use and suPAR is related to the principal pharmacodynamic effects of THC *v.* that of the generic mechanisms related to smoke inhalation remains to be explored. Controlling for tobacco use only modestly affected the strength and magnitude of the association between cannabis use and suPAR in our sample. This supports the hypothesis that generic smoke inhalation effects may not fully explain our observed association. Epigenome-wide association studies have noted similar effects of cannabis use independent of smoking tobacco on methylation status of sites involved in pro-inflammatory pathways (Garrett et al., 2024; Nannini et al., 2023). These investigators observed significant overlap in methylation sites involved in tobacco smoking and cannabis use, therefore, studies disentangling the effects of smoking and cannabis use would be intriguing (Garrett et al., 2024; Nannini et al., 2023). Such studies are now feasible given the changing patterns of cannabis use, particularly in young people (Gunn, Aston, Sokolovsky, White, & Jackson, 2020).

We also did not find any linear associations between cannabis use and IL-6, CRP, or TNF*α* in line with previous studies (Corsi-Zuelli et al., 2022; Doggui, Elsawy, Conti, & Baldacchino, 2021; Okafor et al., 2020). A potential explanation of positive findings in relation to suPAR and negative findings in relation to CRP, IL-6, and TNF*α* may be that suPAR represents a more stable measure of overall immunologic activity (Thunø, Macho, & Eugen-Olsen, 2009). Findings on estimates of the short-term stability of CRP, IL-6, and TNF*α* are heterogeneous (Walsh et al., 2023). Rasmussen et al. (2020) found in a latent class analysis of a general population sample that suPAR may be elevated in the absence of similarly elevated IL-6 and CRP. Rasmussen et al. (2020) hypothesized that this biomarker profile is indicative of systemic chronic inflammation in the absence of acute inflammation. That cannabis use is associated with chronic and not acute inflammation represents another plausible explanation for our findings.

This study has a number of strengths. First, we included a comprehensive array of covariates collected over the span of 24 years of this longitudinal study and we were able to include relevant covariates from gestation. These covariates only reduced the magnitude of the estimates and strength of the association modestly. To our knowledge, this is the first study to show robust evidence of an association between daily cannabis use and systemic chronic inflammation in humans. Our findings are timely and in the context of a changing epidemiological landscape of cannabis use. Associations between legalization of cannabis and increased rates of daily use or dependency have been shown in all recent studies investigating adolescents (Cerdá et al., 2020; Imtiaz et al., 2023; O'Grady, Iverson, Suleiman, & Rhee, 2024; Schuermeyer et al., 2014). In the USA, changes in cannabis policies have been specifically relate to large increases in daily/near daily use, most notably a 15-fold increase between 1992 and 2022 (Caulkins, 2024). THC content in cannabis has also increased over the last number of decades internationally; this has coincided with a possible tripling in transition rates from initiation of cannabis use to disordered use to approximately 30% (Anthony, Warner, & Kessler, 1994; Hasin et al., 2015). In Europe, despite cannabis being largely illicit, potency has continued to increase between 2010 and 2019 (Manthey, Freeman, Kilian, López-Pelayo, & Rehm, 2021). Di Forti et al. (2019) demonstrated two pertinent findings from the EU-GEI study. First, the adjusted incidence of psychosis in Europe is highly correlated with daily use of high potency cannabis in the population (*r* = 0.8). Second, the population attributable fraction of the association between cannabis use and psychosis incidence is above 50% in areas where the prevalence of frequent high-potency cannabis use is elevated (Di Forti et al., 2019). Together these recent research results highlight the urgency of improving the mechanistic understanding of cannabis on health.

There are some limitations with this work. As we only had one measure of suPAR at a single time point, it was not possible to establish a temporal order within the cannabis and suPAR association. We did not analyze data on quantity, chemical content, or method of consumption of cannabis and this may be a limitation. Previous work concluded that frequent cannabis users do not have good knowledge of the content of cannabis they use therefore future studies would benefit from analyzing cannabis used by participants (Kruger, Kruger, & Collins, 2021). We were also unable to analyze whether synthetic cannabinoids could have influenced our results; however self-reported use of synthetic cannabinoids at the time of data collection of this study was low in England and Wales at <1% past year prevalence in adults (Lader, 2015). Unmeasured confounding is another issue, common to all longitudinal studies, and it is possible that our findings could be explained by mental disorders that occurred prior to the onset of cannabis use. It is possible that unobserved patterns within potential confounders such as childhood trauma may account for some of the association seen between cannabis use and suPAR (Rasmussen et al., 2020). It is also possible that there is additional confounding from unmeasured negative prodromal symptoms of psychosis – our measures captured thought interference and positive symptoms of psychosis only. As our study utilized peripheral markers of inflammation, we are unable to conclude that frequent cannabis users experience elevated brain inflammation. Animal models have concluded that peripheral immunomodulatory changes following cannabis treatment are consistent between the periphery and the brain (Moretti et al., 2015). Future work investigating both longitudinal samples and CSF in cannabis users may confirm this. Our study was also not adequately powered to test for interactions with biological sex. In at least one previous study, CB1R has shown to be a dependent factor in the male microglial inflammatory response but not in females (De Meij et al., 2021). There are well-described sex differences in the endocannabinoid system, and psychosis population attributable risk fraction associated with cannabis use is specifically highly elevated in young males (Cooper & Craft, 2018; Hjorthøj et al., 2023). Also of note, a specific mutation in the *PLAUR* gene (rs4760), implicated in elevated levels of suPAR and has been associated with an increased risk of schizophrenia in males, converging with the sex-dependent increased risk of schizophrenia with heavy cannabis use (Hjorthøj et al., 2023; Karagyaur et al., 2024). Another potential limitation in our work is that we only found elevations within a single biomarker of inflammation. The potential reasons for this may be multifold. IL-6 has both pro- and anti-inflammatory actions a determination of its activity may be difficult to ascertain, particularly in general population samples of young people where the incidence of poor health is assumed low. Given the implication of cannabis in neurodegeneration, it is interesting to note that the effects of very high levels of IL-6 may actually prevent impairment of neurogenesis induced by elevated levels of other chemokines (Borsini, Di Benedetto, Giacobbe, & Pariante, 2020; Hirvonen et al., 2012). Ostrowski et al. (2005) in fact showed in experimental models that IL-6 may even attenuate expression of suPAR during endotoxemic challenge – posing questions about the relationship between these biomarkers. A specific issue with TNF*α* is its half-life which is less than 10 min – this may pose sensitivity issues in the context of this study (Held, Hoppe, Cvijovic, Jirstrand, & Gabrielsson, 2019). CRP and suPAR predict health outcomes independently therefore they may be involved in pathophysiological processes (Lyngbæk et al., 2013). A final limitation is the use of a limited number of immune biomarkers within this study. Future studies may benefit from the use of more comprehensive immune-age measures derived from high-dimensional longitudinal data in young people (Alpert et al., 2019).

In summary, our study found that daily/near daily cannabis use is strongly associated with elevated levels of suPAR, a marker of chronic inflammation, at age 24. The relationship between cannabis use and elevated suPAR in particular raise intriguing questions about mechanisms that may underpin the relationship between cannabis exposure; psychotic disorder; and potential roles of frequent cannabis use in oxidative stress, and potential role in chronic diseases in multiple systems (Di Forti et al., 2019; Eugen-Olsen et al., 2010; Haupt et al., 2019; Petersen et al., 2020; Rasmussen et al., 2021).

## Supporting information

Power et al. supplementary material Power et al. supplementary material

## Supplementary material

The supplementary material for this article can be found at https://doi.org/10.1017/S0033291724002848.
