# Supplementary material for: Cannabis use in youth is associated with chronic inflammation
Source: Psychol Med. 2024 Dec 9;54(16):4665–75. doi: 10.1017/S0033291724002848 (PMC11779551; doi:10.1017/S0033291724002848)
Supplement: Power et al. supplementary material [file S0033291724002848sup001.docx]

Supplementary Materials

eTable 1: Sample characteristics within complete case analyses

| Subsample | suPAR | % missing | TNFα, IL-6, CRP | % missing |
| --- | --- | --- | --- | --- |
| N of samples passing quality control | 913 |  | 767 |  |
| *Independent Variables* |  |  |  |  |
| % Daily cannabis use | 4.71 | 0.33 | 4.17% | 0.39 |
| % Weekly/ Monthly cannabis use | 6.9 | 0.33 | 6.91% | 0.39 |
| % <Monthly cannabis use | 20.99 | 0.33 | 20.68% | 0.39 |
| *Covariates* |  |  |  |  |
| Emotional abuse (%) | 20.92 | 12.05 | 20.73 | 12.65 |
| Emotional neglect (%) | 7.34 | 15.55 | 7.3 | 15.91 |
| Physical abuse (%) | 22.78 | 8.87 | 22.55 | 9.39 |
| Childhood sexual abuse (%) | 12.59 | 27.38 | 12.78 | 27.77 |
| Domestic violence (%) | 13.88 | 17.2 | 13.95 | 17.08 |
| Mean BMI (SD) | 24.82 (5.3) |  | 24.89 (5.46) | 1.3 |
| % Past-month non-daily smoking | 17.52 | 0.77 | 17.73% | 0.91 |
| % Daily smoking | 14.24 | 0.77 | 14.08% | 0.91 |
| Median AUDIT-C score (IQR) | 5 (4-70 | 1.64 | 5 (4-7) | 1.8 |
| Sex (% female) | 62.21 | 0 | 62.45% | 0 |
| Mean Age in months (SD) | 294.1 (9.41) | 0 | 293.71 (9.37) | 0 |
| % Low income household in pregnancy | 1.2 | 24.64 | 1.17% | 24.77 |
| Maternal Education, % degree level | 22.56 | 8.65 | 22.95% | 9 |
| % Moderate/Severe Depression | 22.12 | 0.66 | 24.25% | 0.65 |
| % Generalized Anxiety | 29.35 | 0.44 | 30.25% | 0.52 |
| % Psychotic symptoms in last 6 months | 9.64 | 0.88 | 9.99 | 0.78 |

eTable 2:

Multivariate regression analyses of associations between CRP, IL-6, TNFα & cannabis use frequencies in complete cases

| Frequency of cannabis use | Inflammatory Marker | | | | | |
| --- | --- | --- | --- | --- | --- | --- |
|  | Log CRP | | Log IL-6 | | Log TNFα | |
| **Model 1** | **β (CI)** | **P-Value** | **β (CI)** | **P-Value** | **β (CI)** | **P-Value** |
| Less than monthly use | -0.065 (-0.241, 0.111) | 0.469 | 0.034 (-0.134, 0.202) | 0.691 | 0.025 (-0.152, 0.202) | 0.78 |
| Weekly/ Monthly use | 0.007 (-0.272, 0.285) | 0.961 | 0.271 (0.006, 0.537) | 0.045 | -0.135 (-0.415, 0.146) | 0.346 |
| Daily use | -0.174 (-0.526, 0.178) | 0.331 | 0.102 (-0.234, 0.437) | 0.552 | -0.191 (-0.545, 0.163) | 0.29 |
| **Model 2** | **β (CI)** | **P-Value** | **β (CI)** | **P-Value** | **β (CI)** | **P-Value** |
| Less than monthly use | -0.011 (-0.186, 0.163) | 0.900 | -0.011 (-0.181, 0.16) | 0.903 | -0.024 (-0.218, 0.169) | 0.805 |
| Weekly/ Monthly use | 0.106 (-0.164, 0.377) | 0.441 | 0.217 (-0.048, 0.481) | 0.108 | -0.212 (-0.512, 0.088) | 0.166 |
| Daily use | -0.142 (-0.495, 0.211) | 0.430 | -0.119 (-0.464, 0.226) | 0.497 | -0.362 (-0.753, 0.029) | 0.069 |
| **Model 3** | **β (CI)** | **P-Value** | **β (CI)** | **P-Value** | **β (CI)** | **P-Value** |
| Less than monthly use | 0.061 (-0.182, 0.304) | 0.623 | 0.077 (-0.173, 0.326) | 0.547 | 0.108 (-0.158, 0.374) | 0.424 |
| Weekly/ Monthly use | -0.059 (-0.514, 0.396) | 0.799 | 0.222 (-0.246, 0.689) | 0.352 | -0.07 (-0.568, 0.427) | 0.782 |
| Daily use | -0.276 (-0.945, 0.393) | 0.418 | -0.248 (-0.935, 0.439) | 0.478 | -0.483 (-1.214, 0.248) | 0.194 |
| **Model 4** | **β (CI)** | **P-Value** | **β (CI)** | **P-Value** | **β (CI)** | **P-Value** |
| Less than monthly use | 0.083 (-0.16, 0.327) | 0.502 | 0.081 (-0.17, 0.333) | 0.523 | 0.085 (-0.182, 0.352) | 0.532 |
| Weekly/ Monthly use | -0.008 (-0.47, 0.455) | 0.973 | 0.246 (-0.232, 0.724) | 0.312 | -0.139 (-0.647, 0.368) | 0.590 |
| Daily use | -0.148 (-0.825, 0.529) | 0.667 | -0.217 (-0.917, 0.482) | 0.542 | -0.587 (-1.331, 0.156) | 0.121 |

*Each 1-unit increase in the β coefficient is equivalent to a 1 standard deviation increase. Model 1 is unadjusted and contains N=762 subjects. Model 2 is adjusted for age in month, sex, tobacco use & body mass index; and contains N= 749 observations. Model 3 is additionally adjusted for maternal education in pregnancy, weekly family income in pregnancy & AUDIT-C score; and contains N= 414 observations. Model 4 is additionally adjusted for generalized anxiety disorder, moderate to severe depressive disorder, & definite psychotic experiences in the last 6 months; and contains N= 413 observations.

IL-6: Interleukin-6; TNF-α: tumour necrosis factor alpha; CRP: C-reactive protein.

eTable 3: Linear regression analyses of associations between suPAR & past year cannabis use frequencies in complete case analysis

| **Frequency of cannabis use** | **Inflammatory marker** | | |
| --- | --- | --- | --- |
|  | **Log suPAR** | | |
| **Model 1** | **β** | **CI** | **P-Value** |
| Less than monthly use | -0.109 | -0.252, 0.034 | 0.135 |
| Weekly/ Monthly use | 0.028 | -0.201, 0.256 | 0.813 |
| Daily use | 0.662 | 0.389, 0.934 | <0.001 |
| **Model 2** | **β** | **CI** | **P-Value** |
| Less than monthly use | -0.069 | -0.215, 0.077 | 0.356 |
| Weekly/ Monthly use | 0.059 | -0.171, 0.288 | 0.616 |
| Daily use | 0.551 | 0.261, 0.841 | <0.001 |
| **Model 3** | **β** | **CI** | **P-Value** |
| Less than monthly use | 0.043 | -0.143, 0.229 | 0.649 |
| Weekly/ Monthly use | 0.172 | -0.161, 0.506 | 0.311 |
| Daily use | 0.653 | 0.179, 1.127 | 0.007 |
| **Model 4** | **β** | **CI** | **P-Value** |
| Less than monthly use | 0.03 | -0.157, 0.218 | 0.752 |
| Weekly/ Monthly use | 0.126 | 0.215, 0.467 | 0.469 |
| Daily use | 0.608 | 0.129, 1.086 | 0.013 |

*Each 1-unit increase in the β coefficient is equivalent to a 1 standard deviation increase. Model 1 is unadjusted and contains N=910 subjects. Model 2 is adjusted for age in month, sex, tobacco use & body mass index; and contains N= 896 observations. Model 3 is additionally adjusted for maternal education in pregnancy, weekly family income in pregnancy & AUDIT-C score; and contains N= 497 observations. Model 4 is additionally adjusted for generalized anxiety disorder, moderate to severe depressive disorder, & definite psychotic experiences in the last 6 months; and contains N= 495 observations.

sUPAR: soluble urokinase plasminogen activator receptor; FMI: fraction of missing information; CI: Confidence interval
